# Influence of Cemented Carbide Composition on Cutting Temperatures and Corresponding Hot Hardnesses

**DOI:** 10.3390/ma13204571

**Published:** 2020-10-14

**Authors:** Anne Vornberger, Tobias Picker, Johannes Pötschke, Mathias Herrmann, Berend Denkena, Alexander Krödel, Alexander Michaelis

**Affiliations:** 1 Department, Fraunhofer IKTS, Fraunhofer Institute for Ceramic Technologies and Systems Dresden, Winterbergstraße 28, 01277 Dresden, Germany; johannes.poetschke@ikts.fraunhofer.de (J.P.); mathias.herrmann@ikts.fraunhofer.de (M.H.); alexander.michaelis@ikts.fraunhofer.de (A.M.); 2 Institute of Production Engineering and Machine Tools, Leibniz University Hannover, An der Universität 2, 30823 Garbsen, Germany; picker@ifw.uni-hannover.de (T.P.); denkena@ifw.uni-hannover.de (B.D.); kroedel@ifw.uni-hannover.de (A.K.); 3 Institute for Materials Science, Dresden University of Technology, 01062 Dresden, Germany

**Keywords:** hardmetals, cemented carbide, cutting, mechanical properties, hardness, thermophysical properties, thermal conductivity

## Abstract

During metal cutting, high temperatures of several hundred-degree Celsius occur locally at the cutting edge, which greatly impacts tool wear and life. Not only the cutting parameters, but also the tool material’s properties influence the arising cutting temperature which in turn alters the mechanical properties of the tool. In this study, the hardness and thermal conductivity of cemented tungsten carbides were investigated in the range between room temperature and 1000 °C. The occurring temperatures close to the cutting edge were measured with two color pyrometry. The interactions between cemented carbide tool properties and cutting process parameters, including cutting edge rounding, are discussed. The results show that cemented carbides with higher thermal conductivities lead to lower temperatures during cutting. As a result, the effective hardness at the cutting edge can be strongly influenced by the thermal conductivity. The differences in hardness measured at room temperature can be equalized or evened out depending on the combination of hardness and thermal conductivity. This in turn has a direct influence on tool wear. Wear is also influenced by the softening of the workpiece, so that higher cutting temperatures can lead to less wear despite the same effective hardness.

## 1. Introduction

Hardmetals or cemented carbides are composed of tungsten carbide (WC) and cobalt (Co) and are widely used materials for metal cutting tools due to their high hardness and good fracture toughness. Around 65% of produced cemented carbide parts are used as tools in metal cutting [1]. The remaining share splits into tools for mining, oil and rock drilling, as well as tools in wood and construction industries or forming tools and dies. To achieve long cutting tool life and optimal process performance it is of high importance to assess the thermomechanical loads and resulting tool wear phenomena. A large number of factors influence wear in metal cutting, e.g., cutting conditions, workpiece material, tool micro- and macrogeometry, tool coating and tool material properties. From a machining point of view many studies exists, in which the observed tool life is correlated with the applied machining parameters [2]. This makes it possible to predict, e.g., flank and crater wear of tools based on empirical wear rate equations [3]. Newer studies utilize finite element methods (FEM) [4,5], but often the tool material properties, e.g., the mechanical and thermal properties at elevated temperatures, are not known in their entirety, which leads to significant simplifications or to not even including the tool material properties at all in these FEM simulations [6]. Investigations with different cutting conditions, different workpiece materials, as well as uncoated and coated tools, were also carried out [7,8]. However, much less work is published about the wear behavior as a function of the cemented carbide properties. In the study from Saketi et al., the diffusion wear of tools with varying WC grain size and thus also varying hardness values was studied. It was found that the wear rate of both crater and flank wear is clearly influenced by the WC grain size [9]. While hardness and strength are recognized as important material parameters, little attention is paid to thermophysical parameters of cutting tools, such as thermal conductivity. However, they are equally important. During metal cutting, significant heat is generated at the cutting edge and the thermal conductivity of the tool itself significantly influences heat flow regardless of the fact that most of the heat is dissipated within the chip [10]. Thus, it is important to study cemented carbide tools in which both mechanical and thermophysical properties are systematically varied.

Studies on cutting edge preparations showed that tailored roundings can improve performance and increase tool life by influencing the cutting process forces, thermal loads and wear. Tool wear of rounded tools differs significantly from sharp tools due to the varying thermo-mechanical loads. The thermal load is significantly influenced by the cutting edge segment S_α_. This is due to the increase in friction at the flank face and the higher plastic deformation in the shear zone. In return, S_γ_ has a marginal influence. Accordingly, the different temperatures must be taken into account when designing the cutting edge rounding [11,12,13].

However, the wear behavior depends not only on the microgeometry and load, but also on the tool material properties. Therefore, in previous work the relation between cutting edge rounding, cemented carbide tool properties and tool wear behavior was investigated. Wear mechanisms such as flank and crater wear as well as cutting edge chipping depend strongly on the used tool material and cutting edge microgeometry. For example, flank wear can be reduced by increasing cemented carbide hardness or decreasing the cutting edge rounding, while the rake face contact length and thus crater wear can only be reduced by decreasing thermal conductivity of the used tool material [14]. The current study expands this investigation to also include the material properties at elevated temperatures and measurements of the arising process temperatures close to the cutting edge. This allows the interpretation of wear phenomena taking into account hardness as well as thermal conductivity of the used tool materials that are actually present at elevated temperatures.

## 2. Materials and Methods

### 2.1. Fabrication and Characterization of Cemented Carbides

Four different grades with high and low values of room temperature hardness and thermal conductivity were produced and characterized at elevated temperatures in this study. The composition and WC grain size were chosen in such a way that a high and low hardness level of approx. 1900 HV10 and 1400 HV10 and a high and low level of thermal conductivity of approx. 80 W/(m·K) and 40 W/(m·K) are obtained. The sample designations indicate whether the level of hardness (first letter) and thermal conductivity (second letter) are high (“H”) or low (“L”) at room temperature.

Conventional powder metallurgy was used for cemented carbide fabrication. The powder mixtures were prepared using ball milling in n-heptane. The exact compositions and milling time are shown in Table 1. The properties and suppliers of the used powders are listed in Table 2. The dried powder was granulated by sieving and then uniaxially pressed. Sample consolidation was carried out in two steps: first, debindering in hydrogen atmosphere and then sintering for 45 min at 1350 °C and 60 bar Ar pressure using a SinterHIP furnace (FCT Systeme, Frankenblick, Germany). The sintered samples were finish machined to obtain SNMN120408 inserts for the cutting tests.

The hardness was measured according to ISO 3878 [15] using a Vickers indenter and a load of 98 N. Hardness measurements were performed at eight different temperatures between room temperature and 900 °C.

The thermal diffusivity was measured at seven different temperatures between room temperature and 1000 °C using the laser flash analysis method according to DIN EN 821 [16]. The thermal conductivity between room temperature and 1000 °C was obtained by multiplying thermal diffusivity with density and specific heat capacity. The density was measured at room temperature using the Archimedes method according to ISO 3369 [17]. The values for the specific heat capacity as a function of temperature were calculated with the software FactSage (v 7.0, GTT-Technologies, Herzogenrath, Germany) using the Scientific Group Thermodata Europe 2014 database.

### 2.2. Measurement of Cutting Process Temperatures

**Figure 1:**
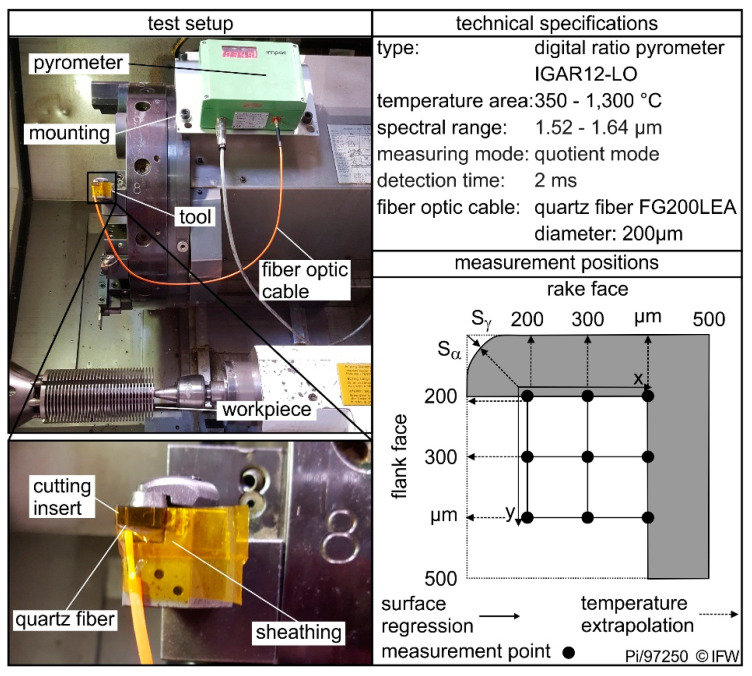
Experimental test setup and technical specifications for the temperature measurements.

Turning tests were carried out to investigate the effects of cutting tool material properties and cutting edge rounding on the occurring process temperatures. The test was performed on a Gildemeister CTX520 Linear machine tool (Gildemeister Drehmaschinen, Bielefeld, Germany) with the manufactured cutting inserts. To ensure a constant chip width and thickness, all tests were run in orthogonal grooving of AISI 4140 with a tensile strength Rm = 928 MPa, a yield strength R_p0.2_ = 690 MPa and a thermal conductivity λ = 42.5 W/(m·K). With the given tool holder, a rake angle γ = −6° and a clearance angle α = 6° was set. The test setup is shown in Figure 1. To investigate the interaction between the cutting tool temperatures, grade properties and cutting edge microgeometry, the four different substrates received a cutting edge preparation. Five different cutting edge microgeometries were produced using abrasive brushing. Afterwards the inserts were coated with a 2 µm layer of TiAlN using a PVD sputter process. The coating is necessary to ensure an industrial relevance of the investigations and to ensure constant friction between workpiece and tool. Since the process temperatures during machining are mainly caused by the frictional heat between the active partners, a different coefficient of friction, due to the different microstructure of the carbides, would be a much greater influencing factor than the carbide properties. Table 3 lists the produced cutting edge microgeometries, as well as the maximum deviation (10%) of the used geometries. In addition to rounding, the contact conditions were varied in form of a continuous and interrupted cut and the cutting speed was varied to v_c_ = 120 m/min and v_c_ = 180 m/min.

The temperature was measured using a ratio pyrometer LumaSense type IGAR12-LO (LumaSense Technologies, Frankfurt, Germany). This uses a quartz fibre-optic cable to record heat radiation from the body and transmit it to the pyrometer. There the radiation is divided by a dichroic beam splitter and the ratio of the present wavelengths is determined. This two-color principle makes it possible to determine the temperature without first determining the emission coefficient ε of the material. At the same time, the pyrometer has a detection time of 2 ms, which also allows highly dynamic temperature changes to be recorded. The disadvantage of this measuring method is the required energy of the thermal radiation, so temperatures could only be recorded from 350 °C upwards. From previous investigations [18,19], it is known, however, that temperatures above 350 °C can be expected during dry steel processing and this measuring method can therefore be used. A quartz fiber FG200LEA from Thorlabs (Newton, NJ, USA) was used to conduct the heat radiation. The fiber had a multi-layer sheathing, which allows the fiber to be used in a temperature and chemical resistant way. Before each measurement, the fiber was polished manually and the sheathing was removed with a special tool. The test setup is shown in Figure 1. To obtain a tool temperature map basically, nine measurement positions for each investigation were used (see Figure 1).

The quartz fiber was positioned by means of previously set grooves in the tool. The grooves were produced by laser ablation on a Sauer Lasertec 40S (Sauer, Pfronten, Germany). The depth, as well as the distance to the rake face, were varied in order to position the fiber accordingly. The measuring points were arranged in a rectangular pattern with an edge length of 0.2 mm. The measurement closest to the cutting edge was performed at a distance of 0.2 mm from the flank and 0.2 mm from the rake face. The geometrical predefined positioning of the measurement points allowed the determination of temperature gradient in the wedge. With a polynomial surface fitting function, the temperature field could be calculated between the nine measured temperatures. This surface function was also used to extrapolate the temperatures towards the rake and flank face [12]. All measurements were repeated once.

The uncertainties in temperature measurement are composed of the device- and setup-specific uncertainties as well as the positioning uncertainty of the optical fibers. According to the manufacturer’s specifications, the ratio pyrometer has a measuring error of ∆U_Pyr_ = 0.7%. In addition, in the used test setup errors in of the groove geometry and the resulting heat accumulation in the cutting wedge occur. According to FE simulations based on the simulations by Bassett [19] this error is ∆U_Geo_ = 6%. To record the positioning error of the optical fiber, three temperature measurements were carried out at constant process parameters. However, for each measurement the cutting insert was changed, and the quartz fiber had to be repositioned. This procedure was repeated for different cutting edge rounding, cutting speeds and measuring positions. The statistical uncertainty of the positioning was 1.7%. Thus, the total uncertainty of the temperature measurement is ∆U_Total_ = 6.3%.

## 3. Results

### 3.1. Cemented Carbide Properties at Elevated Temperatures

**Figure 2:**
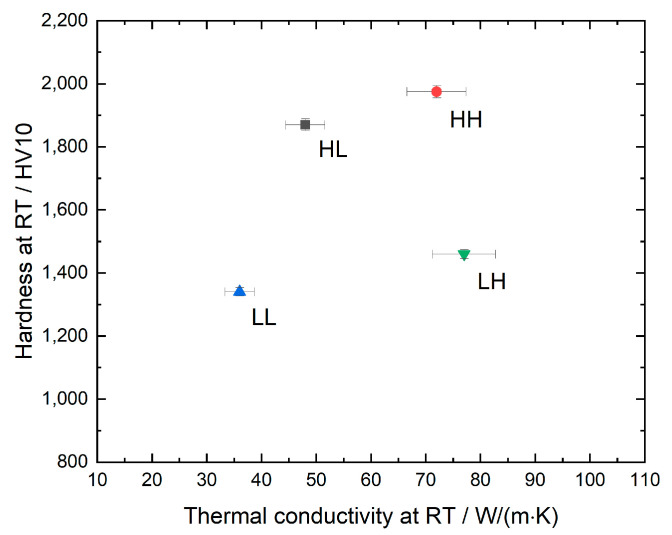
Thermal conductivity and hardness at room temperature.

The measured values for both hardness and thermal conductivity at room temperature (RT) are shown in Figure 2. The first letter in the short designation indicates whether the hardness level at RT is low (“L”) or high (“H”) and the second letter indicates the same for the thermal conductivity.

**Figure 3:**
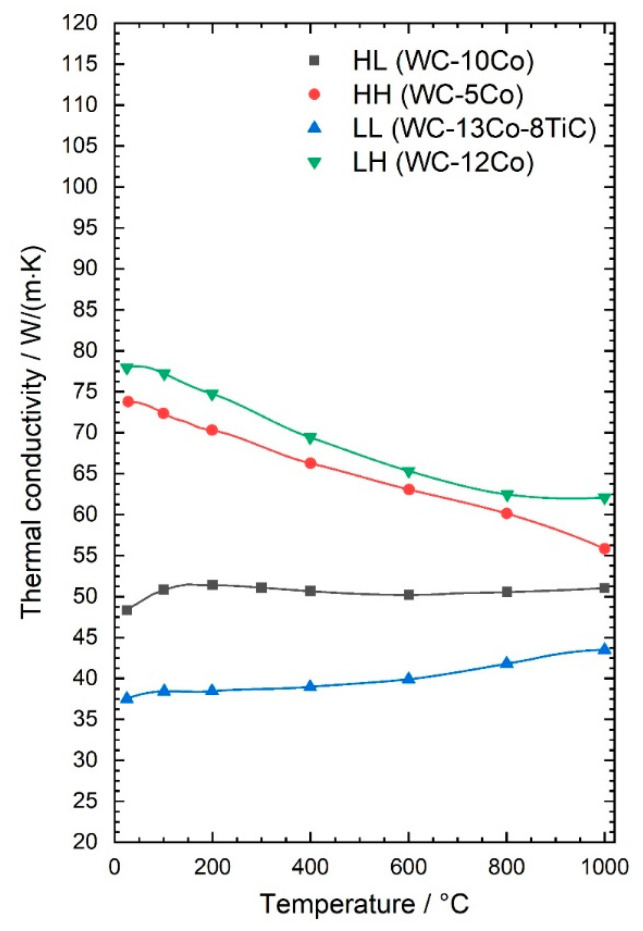
Thermal conductivity of the studied cemented carbides as a function of temperature.

Both, the hardness as well as the thermal conductivity of the grades were measured between RT and 1000 °C. The thermal conductivity λ as a function of temperature is shown in Figure 3. The cemented carbides with high thermal conductivity at RT show a clear decrease of approx. 30% in λ between RT and 1000 °C, while the grades with low thermal conductivity at RT show an increase between 6% to 16%. Consequently, the difference between the high and low level of thermal conductivity gets smaller with increasing temperature. At RT, the cemented carbides with high λ have a 70% higher thermal conductivity and at 800 °C they have only a 30% higher conductivity. Thermal conductivity decreased with increasing metallic binder content and decreasing WC grain size. The difference in thermal conductivity at room temperature diminished with increasing temperature. Heat conduction in solids with metallic conductivity like WC and the metallic binder is caused by the amount and mobility of the free electrons. This is influenced by the nature of the crystal lattice and the disturbance of the lattice by additional atoms or vacancies and especially by the grain boundaries. Therefore, fine grained materials have usually a lower thermal conductivity as the same material with larger grain size [20]. As temperatures rise the mobility of the electrons is reduced by lattice vibration and subsequently the electrical and thermal conductivity decrease. This is the case for materials with high room temperature conductivity. The materials with low grain size, and hence low thermal conductivity, do not show this trend. This is because the thermal diffusivity stays relatively level, but the specific heat capacity slightly increases with increasing temperature. Overall, the curve progressions of cemented carbides with different hardness, but same thermal conductivity are very similar.

**Figure 4:**
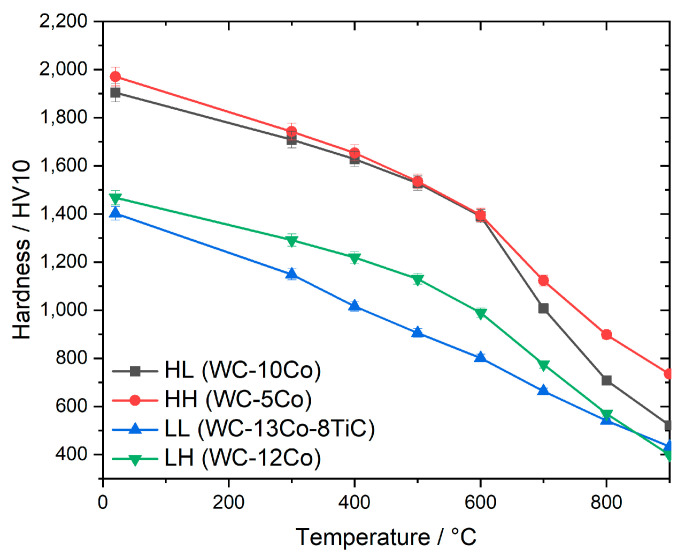
Hardness of the studied cemented carbides as a function of temperature.

**Figure 5:**
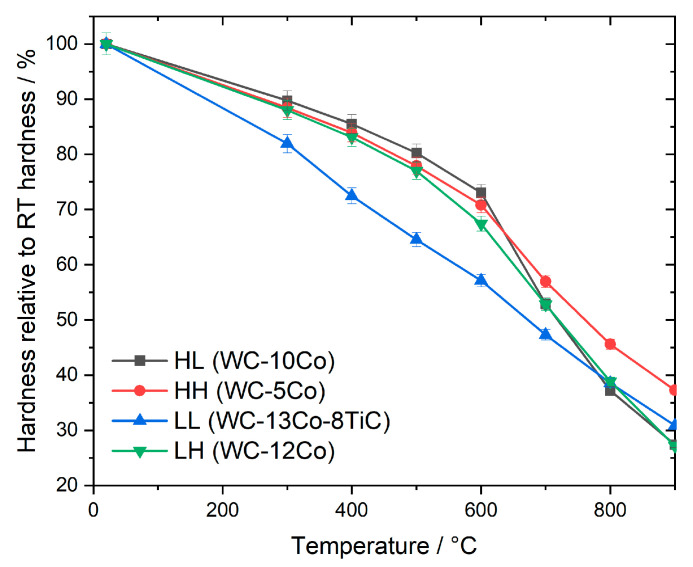
Relative hardness (in relation to RT) of the studied cemented carbides.

The hardness as a function of temperature is shown Figure 4. With increasing temperature, the hardness decreases, but there are differences in the slope of this decrease. To visualize these differences more clearly, the hardness in relation to RT is shown in Figure 5 as a function of temperature.

For the grades with high hardness at RT the curve progression is very similar up to 600 °C. Above this temperature the cemented carbide grade HL shows a very steep decrease in hardness compared to grade HH. Both grades have the same RT hardness but different binder contents as well as different WC grain sizes. Based on previous work, the difference in slope can be mainly attributed to the difference in WC grain size, which is nanosized (0.1 µm) in case of HL and ultrafine (0.3 µm) in case of HH [21].

However, the binder content also plays a role in hot hardness. The grades HH and LH have a comparable WC grain size (0.3 and 0.4 µm, respectively), but different binder content (5% and 12% Co, respectively). Up to 500 °C, the relative hardness (portion of hardness compared to RT at elevated temperature) of these grades is similar. At 600 °C and above, the grade LH with high binder content clearly retains less hardness compared to grade HH. This is due to the high metal binder plasticity in this temperature range. Hence a low metal binder content is very favorable for high hot hardness.

In case of the cemented carbides with the same low hardness at RT differences in hot hardness are already visible at 300 °C. The grade LL with cubic carbide (TiC) addition has a nearly constant decrease in hardness with increasing temperature while in comparison the straight WC-Co grade HL retains higher hardness up to 600 °C. Above 600 °C a steeper decrease in hardness is visible for grade HL which is similar to the grades with high RT hardness.

### 3.2. Cutting Process Temperature during Continous and Interrupted Cutting

**Figure 6:**
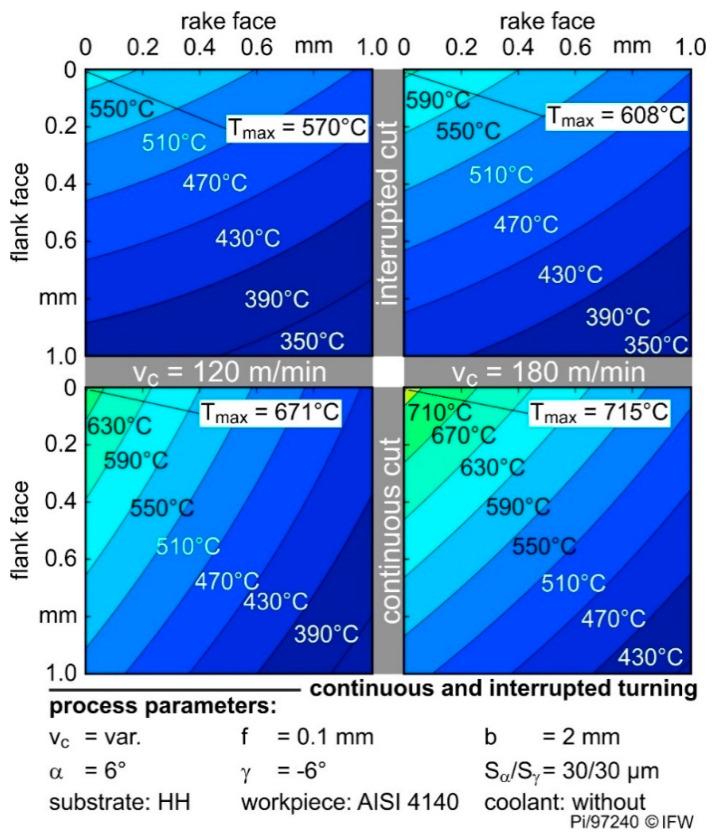
Tool temperatures of different contact conditions and cutting speeds. (The calculated maximum temperatures take the geometry of the cutting edge into account).

The cutting speed and contact condition during machining significantly influence temperatures at the cutting edge in cutting tools. Figure 6 shows the calculated temperature distribution of the HH grade with a symmetrical S_α_ = S_γ_ = 30 µm rounding for continuous and interrupted machining.

The maximum temperature T_max_ always occurs at the cutting edge and temperature decreases with higher distance to the tool-workpiece contact area. By increasing v_c_ from 120 to 180 m/min, the maximum temperature rises from 671 °C to 715 °C, which is an increase of 7%. With increasing cutting speeds, the tool temperatures rise. In this case, the increase in temperature can be explained by the higher relative speed between the chip and the cutting wedge. The higher speed leads to higher frictional energy, which results in the rise of temperature.

A comparable increase in temperature due to the cutting speed also occurs during interrupted machining. However, the temperatures are generally lower than in continuous cutting. At the same cutting speed but interrupted instead of continuous machining, the temperature drops by 16% at 120 m/min and 15% at 180 m/min, respectively. This is due to the periodic change of the tool contact to the workpiece and the associated heating and cooling phase of the cutting edges in the interrupted process. However, the dynamic heating and cooling of the tool tip could not be measured at the individual measuring positions. Due to the distance between the temperature generation zone and the measuring positions, the inertia of the temperature change leads to the recording of the average temperatures. Nevertheless, a significant temperature influence during interrupted machining can be seen. These relationships with regard to cutting speed and contact conditions occurred independently of the cemented carbide used and the applied rounding of the cutting edge.

**Figure 7:**
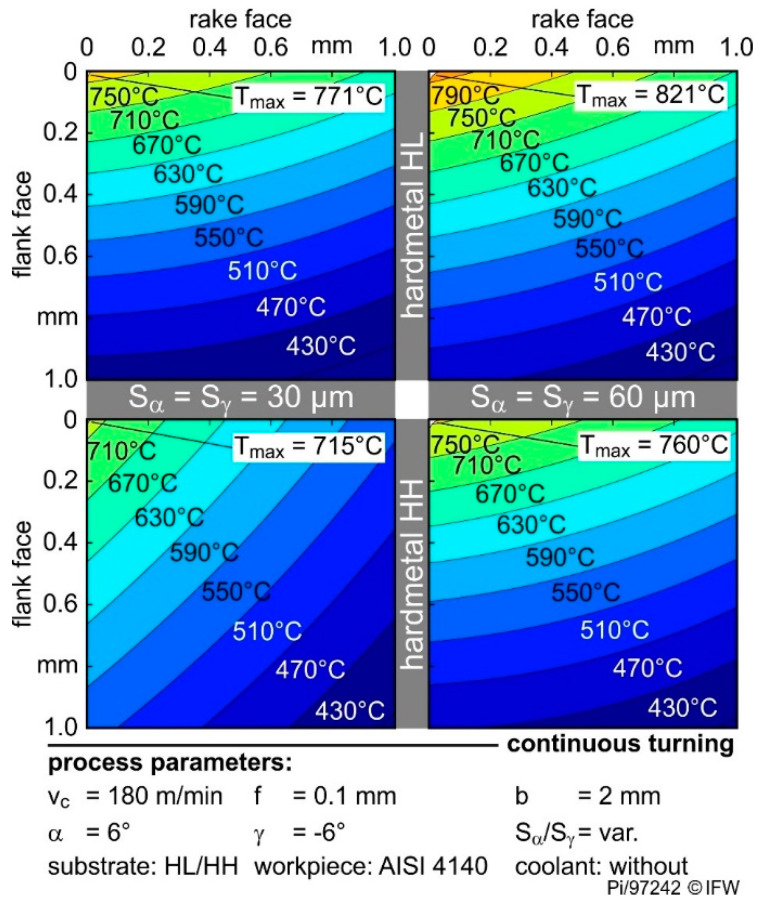
Influence of cutting edge roundings and cemented carbide thermal conductivity on the temperature distribution in cutting tools.

Figure 7 shows the temperature distribution of the HH and HL grades with symmetrical rounding S_α_ = S_γ_ = 30 µm and 60 µm, respectively. Using HL with a low thermal conductivity the maximum tool temperature rises from 771 °C to 821 °C by increasing the cutting edge rounding from 30 to 60 µm. While using the same roundings, the temperature at the cutting edge rises from 715 °C to 760 °C by changing HH material to HL material.

Doubling the rounding increases the cutting edge temperatures in both substrates by 6%. Thus, the influence of cutting edge rounding can be transferred to different cemented carbide properties. The use of larger cutting edge roundings leads to higher maximum temperatures at the cutting wedge. In addition, according to the findings of previous work [11,12,13], the temperatures on the flank face increase with rising cutting edge segment S_α_, respectively, rising cutting edge arc length l_α_.

The higher temperatures are due to the significant increase in the friction, caused by the growing contact length between the material and the cutting edge rounding. However, the influence of thermal conductivity on the temperatures in the cutting wedge is clearly shown in Figure 7. As the thermal conductivity increases from 48 to 72 W/(m·K), the maximum temperature drops by approx. 7% in each case. However, not only the maximum temperatures are affected, but also the temperature distribution. The isotherms are wider at the high thermal conductivity than at the low, which represent lower temperature gradients at a high thermal conductivity. The maximum temperature, as well as the temperature distribution, are results of the different heat conduction in the cemented carbide.

**Figure 8:**
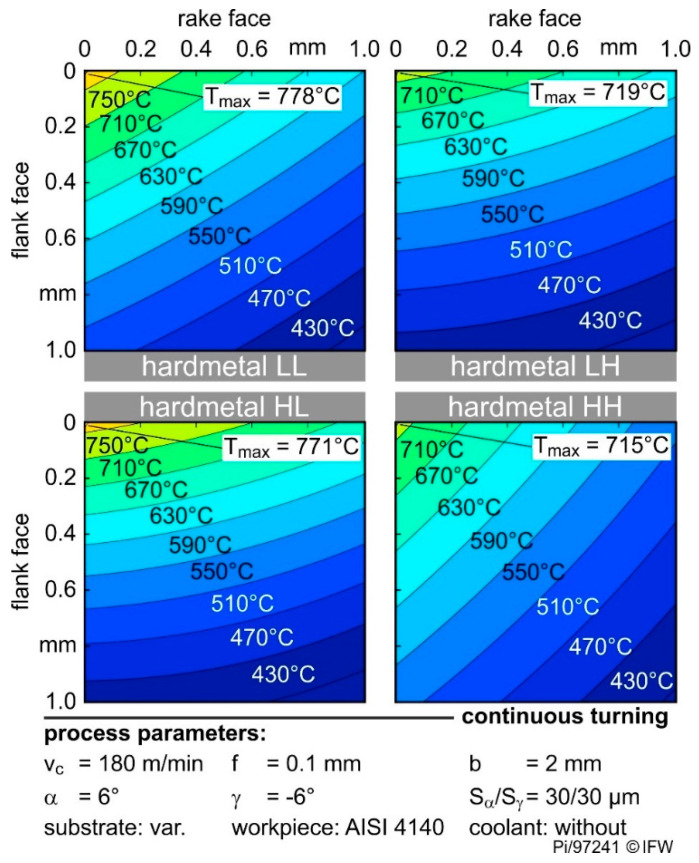
Comparison of the temperature distribution of the used cemented carbides.

This becomes clearer in Figure 8 which shows the temperature distributions of the four grades investigated for one rounding (S_α_ = S_γ_ = 30 µm) and one process parameter combination (continuous cut, v_c_ = 180 m/min). The substrates with similar thermal conductivity but different hardness (LH and HH or LL and HL) have a similar temperature distribution and maximum cutting edge temperature. Additionally, process- and geometry-related influences exist as described above.

## 4. Discussion

### 4.1. Influence of Tool Material Properties on Cutting Process Temperature

**Figure 9:**
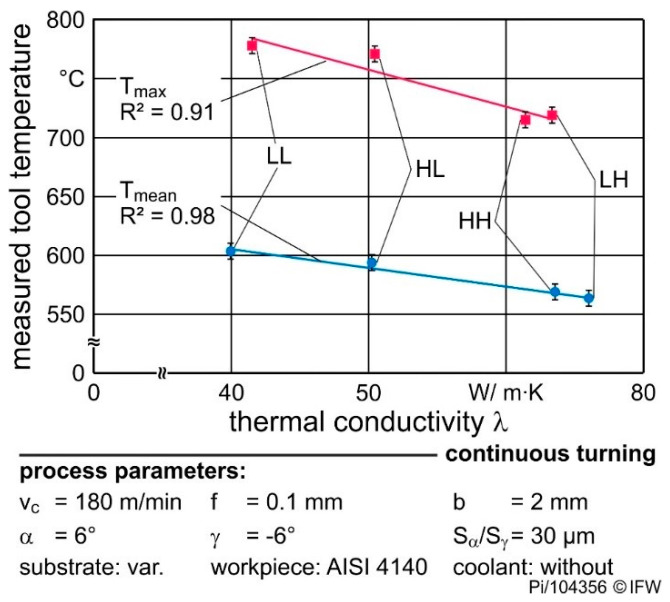
Relation between mean and maximum tool temperatures and the cemented carbide thermal conductivity (continuous cutting, v_c_ = 180 m/min, cutting edge rounding S_α_ = S_γ_ = 30 µm).

Figure 9 shows the relation between the measured tool temperatures and the corresponding thermal conductivities at RT. Both the maximum and mean temperature (nine measurements) decreases linearly with higher thermal conductivity. Measurement of thermal conductivity as a function of temperature showed that the difference between the low and high level of thermal conductivity is not as large at cutting temperature compared to the values at RT. However, the difference is still large enough to cause significant differences in cutting temperature. An increasing thermal conductivity of the cutting tool material is followed by lower maximum temperatures in the wedge and overall lower temperature gradients. With higher conductivity, the heat flow rate from the thermally highly stressed cutting areas increases, which reduces the thermal energy at the contact surface. Consequently, the maximum temperature decreases with higher conductivity. This occurs even though the thermal conductivities of the four cemented carbides change with temperature (Figure 3). The difference between the thermal conductivities is still high enough to ensure a change in the mean and maximum temperature (see Figure 9). As a result of the different thermal loads, the mechanical properties of the tool and workpiece material change differently and affect the occurring tool wear.

### 4.2. Relationship between Cutting Temperature, Cemented Carbide Properties and Tool Wear

The analysis of the hot hardnesses shows a significant decrease in the area above 500 °C. The temperature measurements revealed process temperatures of 700 °C and more during the cutting process. Using the determined dependence of the hardness on the temperature and the calculated temperature of the cutting edge the effective hardness of the cutting edge could be calculated for the different materials and cutting conditions.

**Figure 10:**
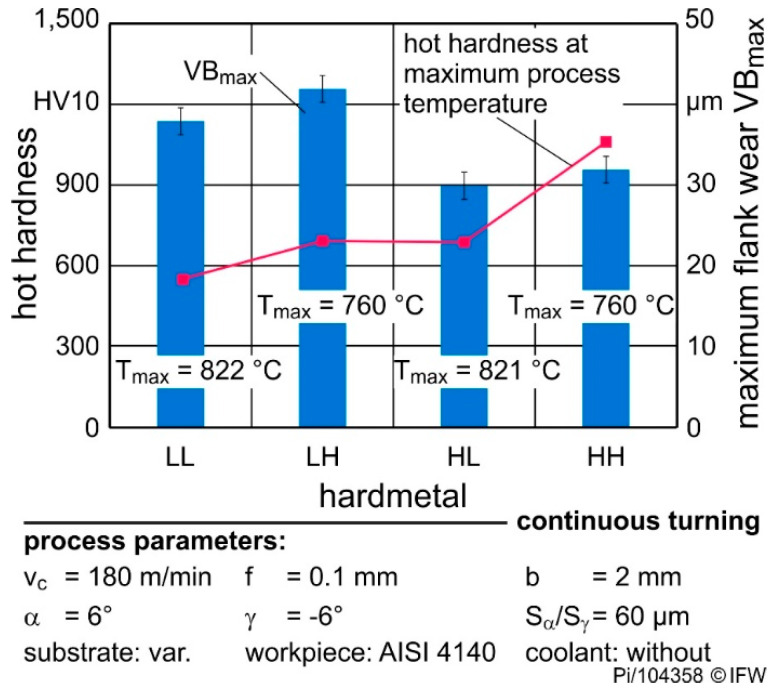
Maximum flank wear and effective hardness (hot hardness) of the used cemented carbides (continuous cutting, v_c_ = 180 m/min, cutting edge rounding S_α_ = S_γ_ = 60 µm, rake face distance = 0.06 µm).

Furthermore, thermal conductivity of the cemented carbide influences the cutting temperature and thus also the effective hardness at the cutting edge. Accordingly, cemented carbides with low thermal conductivity lead to higher cutting temperatures, which reduce the effective hardness of the tool at the cutting edge. This impact of cemented carbide composition and microstructure leads to the remarkable result that grades LH and HL have comparable hardness values at cutting process temperature. As shown in Figure 10 the effective hardness is 688 HV10 und 694 HV10, respectively, in case of v_c_ = 180 m/min and cutting edge rounding S_α_ = S_γ_ = 60 µm, although the difference at RT is approx. 400 HV10.

The opposite is true when comparing the HH and LL materials. With these materials, the difference in effective hardness at the cutting edge increases in comparison with the ambient temperature hardness. These changed tool properties influence directly the occurring tool wear [14].

Depending on the thermal conductivity, the effective hardness at the cutting edge, therefore, changes very differently and differences in room temperature hardness cannot be used to explain the cutting behavior.

During wear investigations the grades had a different maximum flank wear VB_max_ while machining with constant process parameters and microgeometries (see Figure 10). The cemented carbides with higher hardness in the contact area (rake face distance = 0.06 µm), i.e., grade LL and HH lead to approx. 20% less flank wear compared to the grades with low hardness. With higher hardness, the resistance against abrasion increases, leading to lower abrasive wear in form of a lower flank wear width. Nevertheless, the flank wear of HL is 30% lower than LH although both substrates have the same hardness. The current work shows that grades with low thermal conductivity indeed retain less hot hardness compared to high conductivity grades due to the increased cutting temperature. However, the cutting process temperature does not only affect the mechanical properties of the tool material, but also of the workpiece material. Less workpiece softening occurs if the cutting temperature is lower. According to investigations of Brnic et al. the tensile strength of AISI4140 at 700 °C is 15% and at 800 °C 8% of the tensile strength at RT [22]. Regarding these two phenomena, the hot hardnesses of LH and HL are similar, but regarding the tensile strength of the workpiece, respectively, its hardness is 7% lower due to the higher temperatures in the contact area when using the HL substrate. Thus, in case of the grades LH and HL, the workpiece softening could be the dominating factor in respect to the different flank wear.

**Figure 11:**
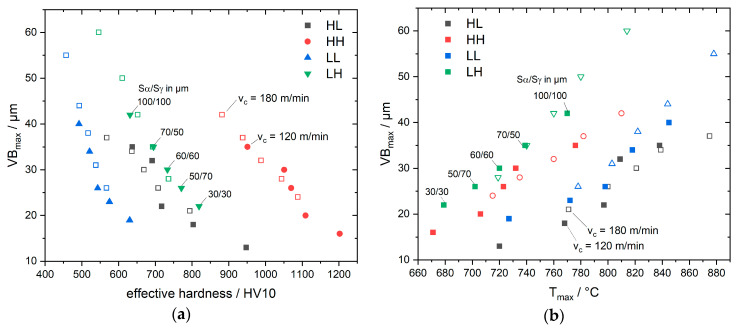
Maximum flank wear VB_max_ as a function of (**a**) effective hardness and (**b**) maximum temperature T_max_ for different cutting edge roundings S_α_/S_γ_ and different cutting speeds v_c_ = 120 m/min (filled symbols) and v_c_ = 180 m/min (unfilled symbols).

Figure 11a,b shows the maximum flank wear VB_max_ as a function of the effective hardness (hot hardness at maximum temperature) and measured maximum temperature, respectively, for all cutting roundings. These diagrams show the interaction between cutting edge rounding, cutting temperature, hot hardness and resulting flank wear, as discussed above, for one cutting edge rounding.

Furthermore, the different process temperatures not only influence the occurring flank wear, but also the rake face contact length and the resulting crater wear [14]. In previous investigations, higher thermal conductivity led to higher contact between the rake face and chip [14]. With the present results, this can be explained by the lower temperatures in the contact area and a resulting lower chip curvature. The lower curvature leads to higher contact length and would result in higher crater wear. A similar relationship was found for the cutting speed, with higher speeds leading to shorter contact lengths. According to the temperature measurements, the temperatures decrease at lower cutting speeds, which in turn lead to higher rake face contact length. Thus, the hypotheses put forward in [14] regarding rake face contact and crater wear could be validated.

## 5. Conclusions

The cutting process temperatures were measured in continuous and interrupted turning with different cutting speeds, cutting edge roundings and four different cemented carbide grades with different room temperature hardness and thermal conductivity. The temperature at the cutting edge was calculated based on measurements of the temperature distribution during cutting. The data showed the strong influence of the thermal conductivity of the cemented carbide on the temperature at the cutting edge. Lower thermal conductivity of the cemented carbide tool increases the cutting process temperature significantly, as well as higher cutting speed and larger cutting edge roundings.

Based on the measured temperature distribution and the measured hot hardness of the different materials the effective hardness at the cutting edge during cutting could be calculated. The effective hardness strongly depends on the thermal conductivity at constant cutting conditions. Depending on the specific room temperature hardness and conductivity values the differences in the effective hardness between the different grades can be amplified or even levelled out. As a consequence, for example, the effective hardness during cutting is lower for tools with lower thermal conductivity compared to grades with higher conductivity and similar hardness. Therefore, differences in room temperature hardness cannot be used to explain the cutting behavior, which takes place at elevated temperatures.

For example, the thermal conductivity influences the resulting contact length between rake face and chip, respectively, the resulting crater wear. With increasing conductivity, the process temperatures decrease, and the contact length increases. In addition, the amount of flank wear is influenced strongly by the effective hardness which itself results from process temperatures and thermal conductivity of the cemented carbides. Higher hardness at elevated temperatures leads to less flank wear due to the increased resistance against abrasion. However, a higher process temperature e.g., due to lower thermal conductivity of the cemented carbide, results in the softening of the workpiece. Thus, a combination of the workpiece softening with a cemented carbide grade which has a high hot hardness (high effective hardness) at these temperatures results in the lowest wear.

The investigation clearly shows that the wear behavior of cemented carbide cutting tools strongly depend on the combination of high temperature hardness and thermal conductivity. Therefore, these parameters are important to understand the cutting behavior and for the selection of the right cutting tool material, geometry and cutting conditions.

## Figures and Tables

**Table 1:** Composition of powder mixtures and milling time.

Designation	WC Powder	WC/wt%	TiC/wt%	Co/wt%	Cr3C2/wt%	VC/wt%	Milling Time/h
HL	WC DN4.0	Bal.	-	10	0.70	0.40	24
HH	WC DS50	Bal.	-	5	0.35	-	12
LL	WC DS250	Bal.	8	13	-	-	12
LH	WC DS80	Bal.	-	12	0.42	-	12

**Table 2:** Properties of sintered cemented carbides at room temperature (RT) [14].

Designation	Sintered WC Grain Size/µm	Hardness at RT/HV10	Thermal Conductivity at RT/W/(m·K)
HL	0.1 ± 0.01	1870 ± 20	48 ± 4
HH	0.3 ± 0.01	1975 ± 20	72 ± 5
LL	0.8 ± 0.02	1310 ± 15	36 ± 3
LH	0.4 ± 0.01	1460 ± 15	77 ± 6

**Table 3:** Used cutting edge microgeometries (calculation of l_α_ and l_γ_ according to [18,19]).

Cutting Edge Segment S_α_/µm	Cutting Edge Segment S_γ_/µm	Edge Arc Length	Cutting Edge Segment S_α_/µm
30 ± 3	30 ± 3	24 ± 2	24 ± 2
60 ± 5	60 ± 5	47 ± 4	47 ± 4
100 ± 8	100 ± 8	79 ± 7	79 ± 7
70 ± 5	50 ± 3	58 ± 4	37 ± 3
